# Prognostic Scores and Azole-Resistant *Aspergillus fumigatus* in Invasive Aspergillosis from an Indian Respiratory Medicine ICU (ICU Patients with IA Suspicion)

**DOI:** 10.3390/jof7110991

**Published:** 2021-11-20

**Authors:** Yubhisha Dabas, Anant Mohan, Immaculata Xess

**Affiliations:** 1 Department of Microbiology, All India Institute of Medical Sciences (AIIMS), New Delhi 110029, India; yubhi.aiims@gmail.com; 2 Department of Pulmonary Medicine and Sleep Disorders, All India Institute of Medical Sciences (AIIMS), New Delhi 110029, India; anantmohan@yahoo.com

**Keywords:** invasive aspergillosis, respiratory medicine ICU, severity scores, galactomannan antigen, mortality, azole-resistant *Aspergillus fumigatus*, India

## Abstract

**Objective:** To assess the effectiveness of three general prognostic models (APACHE II, SAPS II, and SOFA) with serum galactomannan antigen in a clinically suspected invasive aspergillosis (IA) subpopulation admitted to a respiratory medicine ICU and to identify azole-resistant *Aspergillus fumigatus* (ARAF) cases. **Methodology and Results:** A total of 235 clinically suspected IA patients were prospectively enrolled and observed 30-day mortality was 29.7%. The three general models showed poor discrimination assessed by area under receiver operating characteristic (ROC) curves (AUCs, <0.7) and good calibration (*p* = 0.92, 0.14, and 0.13 for APACHE II, SAPS II, and SOFA, respectively), evaluated using Hosmer–Lemeshow goodness-of-fit tests. However, discrimination was significantly better with galactomannan values (AUC, 0.924). In-vitro antifungal testing revealed higher minimum inhibitory concentration (MIC) for 12/34 isolates (35.3%) whereas azole resistance was noted in 40% of *Aspergillus fumigatus* isolates (6/15) with two hotspot cyp51A mutations, G54R and P216L. **Conclusions:** Patients diagnosed with putative and probable IA (71.4% and 34.6%, respectively), had high mortality. The general prognostic model APACHE II seemed fairly accurate for this subpopulation. However, the use of local GM cut-offs calculated for mortality, may help the intensivists in prompt initiation or change of therapy for better outcome of patients. In addition, the high MICs highlight the need of antifungal surveillance to know the local resistance rate which might aid in patient treatment.

## 1. Introduction

Invasive aspergillosis (IA), a known condition of immunocompromised patients has long been accepted as an emerging opportunistic infection in critically ill patients [1,2,3]. The advent of fungal infections in intensive care units (ICUs) has been attributed to the advances in life-support systems, extensive use of broad-spectrum antibiotics and invasive devices, and an increase in the susceptible patient population [4]. However, the delay in IA recognition in these patients can be due to the diagnostic limitations in ICU settings [1,2,3]. The classical risk factors for IA are inapplicable for critically ill patients as many of these patients have competent immune systems [3]. It is a serious problem in ICUs as it is associated with very high mortality (>80%) [5,6]. The non-invasive galactomannan antigen (GM) test still needs validation in critically ill patients but is otherwise very useful in the neutropenic patient population. There is also a lack of data on the applicability of this diagnostic test on the optimal treatment/outcome for these ICU-admitted IA-suspected patients. There are few outcome studies where, depending on the patient population and the infecting organism, attributed mortality rate has been calculated [7]. There are illness scoring systems which have been developed and validated for different ICU subpopulations including Acute Physiology and Chronic Health Evaluation (APACHE) II, Sequential Organ Failure Assessment (SOFA), and Simplified Acute Physiology Score (SAPS) II score. These scoring systems can potentially help the physicians to predict clinical outcomes for infection especially the ones associated with high morbidity and mortality.

The current IDSA (Infectious Disease Society of America) treatment guidelines for invasive aspergillosis (IA) detail antifungal ancillary treatments and also the duration of treatments. Voriconazole is the drug of choice for primary therapy in IA (especially with cases of invasive pulmonary aspergillosis) whereas liposomal amphotericin B, caspofungin, and posaconazole are to be preferably used as the salvage therapy drugs [8]. However, these guidelines are not strictly followed in intensive care units (ICUs).

In 2015, experts discussed the treatment options in clinical cases from regions where azole-resistant *Aspergillus fumigatus* are rampant (>10%) in the environment and suggested use of voriconazole monotherapy in azole-susceptible isolates and in cases of unknown susceptibility, owing to culture negativity, the initial therapy was suggested to be liposomal amphotericin B for 2 weeks later de-escalated to voriconazole or posaconazole if the patient showed good clinical response with the former. However, all experts had consensus on the use of national epidemiology and local susceptibility data to guide the choice of treatment [9].

In the present study, we identified the different factors associated with mortality and evaluated and compared the effectiveness of established general scoring systems (APACHE II, SAPS II, and SOFA) and the GM test in the prediction of ICU mortality of IA-suspected patients. We also evaluated two standard microbroth dilution methods of antifungal susceptibility testing including the Clinical Laboratory Standards Institute (CLSI)-approved CLSI M38-A2 and the European Committee on Antimicrobial Susceptibility Testing (EUCAST)-approved EUCAST Edef 9.3 for the antifungal susceptibility patterns from IA cases in our respiratory medicine ICU.

## 2. Materials and Methods

### 2.1. Patient Population and Study Settings

This prospective study was conducted on the patients with clinical suspicion of IA cared for in the medicine ICUs of a tertiary care hospital from August 2013 to August 2017. Patients clinically suspected of IA displaying at least one of the following host factors were enrolled in the study: hematologic malignancy; cancer and receiving chemotherapy within the last 3 months before admission, with or without neutropenia; chronic obstructive pulmonary disease (COPD); transplant recipient (hematopoietic/solid organ); chronic granulomatous disease (tuberculosis); other immunocompromised state (inherited immunodeficiency, child C cirrhosis, or HIV etc.); steroid use: at least 4 mg methylprednisolone (or equivalent) a day for at least 7 days in the 3 weeks before admission or during the course of the ICU stay for at least 5 days or a cumulative dose of at least 250 mg of methylprednisolone (or equivalent) in the past 3 months before enrolment; recipient of any other immunosuppressive treatment (tacrolimus, cyclosporine, methotrexate, cyclophosphamide etc.); or microbiological evidence of *Aspergillus* infection during the stay in ICU (any positive culture or two positive circulating galactomannan tests) (data not shown). In addition, eligible patients could only be enrolled if they had at least two of the following three features: fever refractory to at least 3 days of appropriate antibiotics or fever relapsing after a period of defervescence of at least 48 h while still receiving antibiotics; clinical signs and/or symptoms suggestive of invasive mycosis: pleuritic chest pain or physical finding of pleural rub, or one of the following symptoms of lower respiratory tract infection (new sputum secretions, dypsnea, or hemoptysis); or development of new pulmonary infiltrates on chest X-ray. The sole exclusion criterion was clinical suspicion of IA before ICU admission.

The study was approved by ethics committee of the institute. The consent forms for minor/incapable participants were obtained by their LAR, i.e., legally accepted representatives (e.g., mother, father, children, or grandparents).

Patients were classified into different groups according to AspICU diagnostic criteria [10]. The clinically suspected patients satisfying the inclusion criteria were included. However, the non-compliant cases were categorized as no invasive aspergillosis (No IA). The clinical details of the patients were recorded and the 30-day mortality to the day of admission was also monitored. Only the patients for whom the complete details were obtained were included in the statistical analysis.

### 2.2. Galactomannan Antigen

We performed the galactomannan antigen (GM) assay using Platelia kit (Bio-Rad, Marnes-la-Coquette, France). Serial serum samples (day 0: the day of enrolment of patient in the study and day 7: the 7th day after the enrolment) were obtained for all the patients who were clinically suspected of IA for a uniform GM analysis as per the physician’s recommendation. According to the defined threshold in neutropenic patients, our threshold to define a positive GM was an optical density (OD) > 0.5. For the determination of the best predictive cut-offs for IA mortality, a receiver operating characteristic (ROC) curve was constructed for GM. The relevant summary diagnostic parameters, including sensitivity, specificity, positive, and negative predictive values (PPV and NPV), were calculated.

### 2.3. ICU Scoring Systems

The Acute Physiology and Chronic Health Evaluation II (APACHE II), Simplified Acute Physiology Score II (SAPS II), and Sequential Organ Failure Assessment (SOFA) scores were calculated based on the worst values recorded during the first 24 h after admission. All enrolled patients were followed during their ICU stay and up to 30 days from the day of their ICU admission, and the outcome was recorded as survivors or non-survivors. The online combination ICU Mortality Calculator (APACHE II, SAPS II, and SOFA scores to predict hospital mortality; http://clincalc.com/IcuMortality/Default.aspx) was used to calculate the corresponding score for each patient. Further analysis of SOFA predicted mortality was done only after taking the mean of the mortality range obtained for the individual patient score as the regression formula to calculate predicted death rate directly with string variable was not possible. The Hosmer–Lemeshow goodness-of-fit test was used to evaluate the agreement between the observed and predicted mortality and was compared with a χ2 test. With this test, a *p* value greater than 0.05 indicated a good fit for the model. Standardized mortality ratios (SMRs) with 95% confidence intervals (95% CIs) were calculated using Mid-P exact test. An SMR greater than 1.0 implies that a prognostic score is an underestimate of the observed mortality, whereas an SMR less than 1.0 is an overestimate of the observed mortality.

### 2.4. Antifungal Susceptibility Testing

Antifungal susceptibility testing was carried out by standard broth microdilution assays using the Clinical Laboratory Standards Institute (CLSI)-approved standard M38-A2 and the European Committee on Antimicrobial Susceptibility Testing (EUCAST) (EDef 9.3) guidelines for molds [11,12]. For CLSI methodology the proposed epidemiological cut-offs (ECVs) were followed [13]. For EUCAST methodology the breakpoints were followed as per the clinical breakpoints v8.1 published on EUCAST website, for amphotericin B, itraconazole, and voriconazole, ≥2 μg/mL; for the remaining drugs, i.e., posaconazole, caspofungin, and micafungin, ≥0.25 μg/mL [14,15]. The readings were taken visually for endpoint by both the methodologies followed except for echinocandins. For caspofungin and micafungin, the MECs (minimum effective concentrations) were calculated and were the lowest echinocandin concentration in which abnormal, short, and branched hyphal clusters were observed microscopically in contrast to the long, unbranched, hyphal elements which were seen in the growth control well. To avoid any errors all assays in the study were run in triplicate.

In this study, cyp51A mutations were sought in all *A. fumigatus* isolates using forward primer P450A1 [16] and reverse primer CYP3 R [17] yielding a PCR product of ~1500 bp.

### 2.5. Statistical Analysis

Continuous variables are presented as either mean (± SD) or median with interquartile range in case of skewed distribution. They were normally distributed and the Student’s t-test was used. The categorical variables were expressed as numbers and percentages of the group from which they were derived. The chi-square test and Fisher’s exact test were used to compare categorical variables as appropriate. Socio-demographic clinical characteristics and risk factors were evaluated by univariate and multivariate analysis. These were entered into a logistic regression model for calculation of unpaired and paired Odds ratios (ORs). The ORs were are given with 95% confidence intervals (CIs). A cutoff of *p* ≤ 0.05, two tailed, was significant for all statistical analysis.

For the determination of the best mortality predictive score system, receiver operating characteristic (ROC) curves were constructed. The areas under the curve (AUCs) were estimated to analyze the discriminatory power. Comparisons between AUCs were performed using the method of Hanley and McNeil [18]. All estimations were reported with 95% confidence intervals (CI).

All statistical analysis were done using STATA version 9 (StataCorp. 2005. Stata Statistical Software: Release 9. College Station, TX: StataCorp LP) except for antifungal data where the two techniques were statistically analyzed with Statistical Package for the Social Sciences software (version 16.0; SPSS S.L., Madrid, Spain).

## 3. Results

### 3.1. Patient Characteristics and Mortality Rate

Table 1 gives the main demographic and clinical characteristics of the 235 patients in the study and the underlying conditions, if present. Serial serum galactomannan antigen (GM) testing was done for all the patients enrolled. According to the AspICU criteria, one was proven IA (died), 21 (8.9%) were putative IA (15 died, 71.4%), 12 (5.1%) were colonized (11 died, 91.7%) and 201 (85.5%) were categorized as No IA cases (43 died, 23.4%) (data not discussed further).

In this cohort of 235 patients, after being monitored for a median period of 14 days interquartile range (IQR) [7,8,9,10,11,12,13,14], 70 of 235 patients died (mortality rate: 29.7%) and 40.4% of the patients (95/235) were on mechanical ventilation with a median duration of 5 days (IQR, 2–11).

The summary of clinical characteristics of the study population on the day of ICU admission and during the ICU stay is shown in Table 2. However, there were only three patients with cardiovascular manifestations and all three died during their ICU stay. The mortality rate was 50% for six liver manifestation and two disseminated intravascular coagulation cases.

On multivariate analysis, variables that were found to be significant predictors of mortality were hematological malignancy (OR 2.14, 95% CI 0.57–8.02), high temperature ≥ 39.4 °C (OR 1.91, 95% CI 0.35–10.28), neutropenia (OR 2.46, 95% CI 0.61–9.97), mechanical ventilation (OR 1.44, 95% CI 0.52–3.97), dialysis (OR 2.17, 95% CI 0.32–14.49), *Aspergillus* culture positivity (OR 7.68, 95% CI 1.42–41.32), and significant galactomannan Ag cut-off index value (≥1.04) for mortality (OR 37.93, 95% CI 13.00–110.71).

### 3.2. Galactomannan Antigen Testing

**Figure 1:**
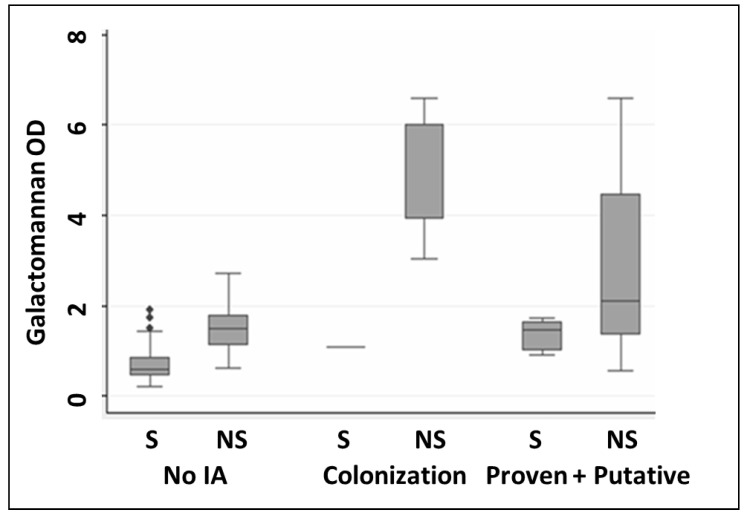
Boxplot characterization of the median galactomannan OD index values categorized using AspICU criteria. (where OD: optical density; S: survivors; NS: non-survivors; IA: Invasive aspergillosis).

Serial serum GM (days 0 and 7) was measured in all (*n* = 235) patients. Following the AspICU criteria, the median GM value for proven and putative was 2.09, 6 for colonization and 1.48 for the cases described as no IA (Table 3) (Figure 1).

**Figure 2:**
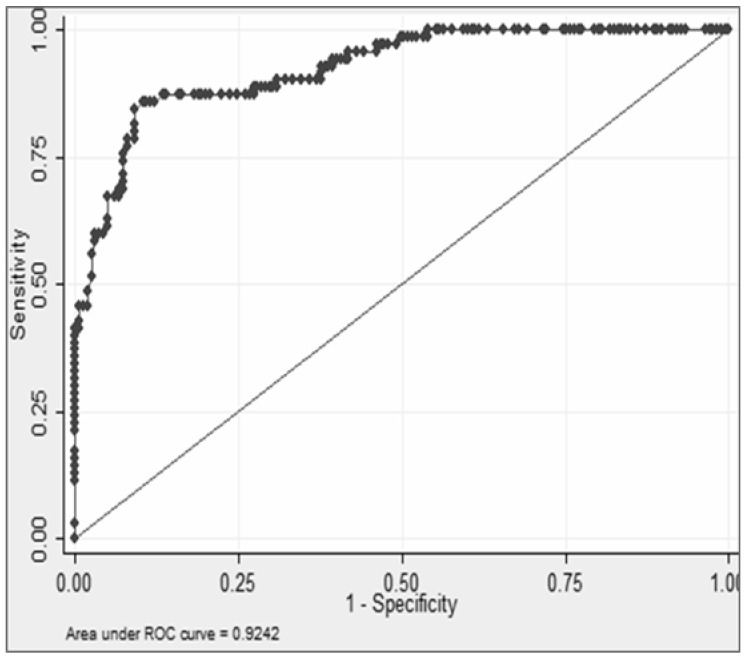
Calculated galactomannan antigen OD index cut-off for mortality with area under the curve (AUC).

The GM cut-off for mortality was found to be ≥1.04 with an AUC of 0.942. The sensitivity, specificity, NPV, and PPV were 87.14, 86.67, 94.08, and 73.49, respectively, (Figure 2).

### 3.3. Performance of the ICU Scoring Systems and Significant GM Cut-Off for Mortality

A comparison of the different ICU scoring systems for the disease severity and estimated mortality in clinically suspected IA subpopulation (Table 4) showed that all three ICU scoring systems had statistically significant differences between survivors and non-survivors (*p* < 0.05) and the unadjusted and adjusted odds ratios also showed significant values (OR > 1).

The mean and median of APACHE II-and SAPS II-score-predicted mortality were significantly higher for the non-survivors (*p* < 0.05) with significant unadjusted and adjusted odds ratio (OR > 1) (Table 5).

**Figure 3:**
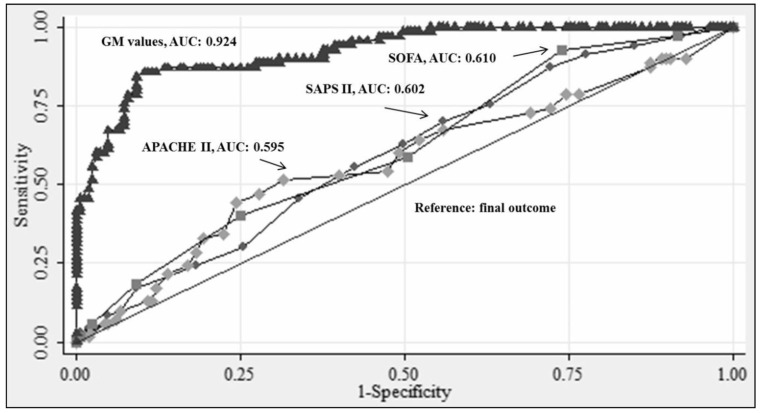
Area under (AUC) receiver operating characteristic (ROC) curves of severity scores (APACHE II, SAPS II, and SOFA) and galactomannan (GM) values. (where APACHE II: Acute Physiology and Chronic Health Evaluation II; SOFA: Sequential Organ Failure Assessment; SAPS II: Simplified Acute Physiology Score II).

All scoring systems displayed similar ROC curves with no significant difference in the AUCs calculated. On the basis of AUCs, none of the scoring systems exhibited excellent discriminatory power (AUCs < 0.7) (Figure 3 and Table 5).

The evaluation of the goodness-of-fit for all three scoring systems showed good calibration (*p* > 0.05 for every score), as shown in Table 5. According to the goodness-of-fit findings, APACHE II (*p* = 0.92) had better calibration than did SAPS II (*p* = 0.13) and SOFA (*p* = 0.14). All three scoring systems tended to give underestimates of mortality (SMR, >1.0); however, the difference between actual and estimated mortality was lowest for APACHE II (SMR, 1.20; *p* < 0.001) followed by SAPS II (SMR, 1.46; *p* < 0.001) and the highest was seen with SOFA score calculations (SMR, 2; *p* = 0.78) (Table 5).

### 3.4. Antifungal Susceptibility Testing

Sample and culture distribution from this cohort of patients describes the isolation of 34 *Aspergillus* sp. (Supplementary Table S1). In-vitro antifungal resistance (irrespective of class of drugs) was seen in 12/34 isolates, 35.29% (Table 6). Seventy five percent (*n*, 9) of the total 12 resistant isolates showed high MICs to the azole group of drugs.

Azole resistance in *A. fumigatus* was noted in high number of isolates 6/15, 40% (of which one was a colonizer strain). Four of these six isolates had shown high MICs to itraconazole, and two showed cross-resistance with itraconazole combined with voriconazole or posaconazole. The cyp51A mutations were also observed in these isolates (Table 7).

## 4. Discussion

Infections are common in ICU patients and are mostly of bacterial origin [19]. However, there is rising concern over opportunistic fungal infections in ICU. The most common fungal infection reported in ICUs is *Candidiasis* followed by *Aspergillosis* [20]. Invasive aspergillosis is mainly studied in neutropenic hematological malignancy cases and is often underestimated in ICU population because of lack of establishing confirmed diagnosis [21]. It is associated with a high mortality rate of 60–80% [1,6]. However, from the total cohort in this study we found a low mortality rate of 29.7%. Since all the consecutive suspected IA cases were included for analysis because of lack of a standard definitive diagnostic criteria in ICU and a lack of possibility of obtaining invasive samples (biopsy, bronchoalveolar lavage) from our patients. As per the different diagnostic criteria applied, the mortality was high for probable cases (35.5%) and was even higher for putative cases (71.2%). A low mortality (37–40%) for IA in ICU has been reported in few studies [22,23]. This may be multifactorial including heterogeneity of the enrolled patients or lack of a defined clinical subset of patient population or inclusion of all suspected cases and shorter duration of follow-up.

The most common risk factors for IA mortality in ICU include: age, bone marrow transplant, mechanical ventilation, renal replacement therapy, cirrhosis, COPD, solid organ malignancy, HIV infection, malnutrition, postsurgical patients, and those on immunosuppressive therapies [1,3]. In our study, by adjusting all the confounding factors, predictors of poor outcome were found to be hematological malignancy, high temperature (>38 °C), low neutrophil counts (≤500/mm^3^), *Aspergillus* culture positivity, high GM cut-off (≥1.04) and for the duration of ICU admission neutropenia, and mechanical ventilation (Table 1 and Table 2).

Global data suggest that higher GM values are often associated with high mortality even in ICU population [24,25,26]. In this study, high GM values were found statistically significant in association with mortality (*p* < 0.001) (AUC: 0.924).

About 30 years ago, for the ICU patient population, the first outcome prediction scores were calculated [27]. Over time, new predictive models have been developed and with specific aims, for example, the Acute Physiology and Chronic Health Evaluation (APACHE) and Simplified Acute Physiology Score (SAPS) were general outcome prediction models; the Multiple Organ Dysfunction Score (MODS) and Sequential Organ Failure Assessment (SOFA) were organ dysfunction scores; and to assess nursing workload the Therapeutic Intervention Scoring System (TISS), etc., were developed [28]. These scoring systems have been used for different ICU subpopulations including cancer, COPD, acute respiratory distress syndrome (ARDS), and mechanically ventilated patients [7,25,29,30,31]. Validation is crucial before the routine application of any mortality predictive model in a patient subpopulation, if different from the one originally used for model development. In this study, we have tested the performance of the three most commonly used scoring systems irrespective of the definite diagnosis of the disease, to ensure the intensivist of the continued accuracy of these scoring systems in the suspected IA subpopulation of ICU. Previously, the general outcome score APACHE II has been used to predict IA (putative or probable vs. colonization)-associated mortality in ICU [1,4,5] and also SOFA scores calculated for the day of positive *Aspergillus* culture have been reported as a significant predictor of mortality [1].

On univariate and multivariate analysis, all the three scoring systems were good predictors of mortality (unadjusted and adjusted OR > 1). The discriminatory power of the three scoring systems was poor (AUCs < 0.7). However, the predicted mortality calculated from APACHE II scores was the best among the three models (expected mortality: 24.6% vs. observed mortality: 29.7%). All scores had acceptable calibration (*p* > 0.05), although the statistical significance for the Hosmer–Lemeshow goodness-of-fit tests were marginal for SAPS II (*p* = 0.13) and SOFA (*p* = 0.14) whereas exceptional for APACHE II (*p* = 0.92) followed by GM values (*p* = 0.52). The SMRs for three scoring models, were best for APACHE II (1.2) followed by SAPS II (1.46) and then SOFA (2) scores.

Even though it seems low, the ICU mortality of our cohort does not differ from mortality reported in other published similar studies, if we only looked at the definite cases of IA as in those studies. An interesting finding of our analysis is the performance of galactomannan antigen as a predictor of mortality. On univariate and multivariate analysis, it was the best predictor of mortality (unadjusted and adjusted OR > 100). The discrimination was excellent (AUC, 0.924) with good calibration (*p* = 0.52) which was preceded by only APACHE II (*p* = 0.92). This may be due to the well-known weakness of the effect of sample size on Hosmer–Lemeshow goodness-of-fit on the measured calibration which does not affect discrimination.

In vitro antifungal resistance of 35.29% was relatively higher than the reported data. Cross-resistance was also seen in two isolates being resistant to multiple drugs. Previous data suggests drug resistance among triazoles is minimal with posaconazole <1% [32]. Incidence of drug resistance with itraconazole and voriconazole is 4.2–7% [33,34] and <3.5% [35], respectively. Overall, multi-azole drug resistance has been reported in up to 1.7%–6% of patients [34]. Cross-resistance patterns are closely linked with the position of the mutation in the cyp51A gene [16,32,33,36].

Itraconazole resistance (5–8%) has increased over time [33]. Reports published from Asia demonstrate environmental resistance mechanism in *A. fumigatus* isolates from two highly populated countries in Asia, i.e., China and India and also from the neighboring Middle East [37]. In a study from Netherlands, 94% of 32 itraconazole-resistant isolates, had the same two alterations: an L98H-amino acid substitution in cyp51A and duplication in the promoter region [34].

The mechanisms of azole resistance in the species have been extensively studied in Western world and the results are alarming and pose a warning for the regular surveillance worldwide. However, scanty data had been published from India focusing on isolates recovered from deadly disease (IA). The only reports until now from India were two studies from the same center where in one study, two, and in another, 12 triazole-resistant isolates were reported and majorly had TR/L98H mutations [37,38]. The same authors had also done pioneer resistance surveillance in the environmental isolates and reported major TR/L98H mutations in 44/630 azole-resistant *A. fumigatus*, [39] they also reported the first TR46/Y121F/T289A mutations in 6/1210 resistant environmental isolates [40].

Overall in this study, the two hotspot mutations leading to azole resistance in *A. fumigatus* were G54R and P216L. In India, these mutations have previously been reported from immunocompromised hosts [41] and a substitution from glycine to glutamic acid has been reported at codon 54 (G54E) in an environmental [39] and clinical strain [37].

Geographically the triazole resistance varies in *A. fumigatus* clinical isolates as it has been reported <1–3.6% in the USA [42,43], 4% in China [44], 2% in India [37], 4.5% in Kuwait [45], and 11% in Japan [46]. Few studies from Europe, Latin America, North America, Middle-East Asia, South-East Asia, and Oceania are listed in Supplementary Table S2 clinical (irrespective of patient groups) and environmental, where the prevalence and mutations leading to azole resistance are mentioned.

The main strength of this study is the analysis of an emergent subpopulation of ICU patients, clinically suspected of IA for accuracy of mortality prediction by otherwise non-specific scoring systems with the evaluation of galactomannan antigen as a marker for predictor of mortality. However, the study was limited by its small size. Second, it was physician-driven (based on clinical suspicion of IA) in a cohort of critically ill patients at particular high risk which gave us an excessive inhomogeneity of patients because of which we could not define an underlying group for our patients. Third, lack of invasive samples (biopsy, BAL) owing to the severely deranged vitals of the patients and the lack of autopsies post mortem may have caused lower numbers of proven case ratios in an otherwise high preponderance of IA in respiratory medicine ICUs.

## 5. Conclusions

The mortality rate for confirmed IA cases in ICU is very high. Out of the general outcome scores, APACHE II can be used to predict mortality for the defined IA suspected ICU subpopulation, as the observed outcome (29.7%) matched the expected mortality (24.6%). The increase in numbers of azole-resistant *A. fumigatus* (ARAF) reported from ICUs has led to awareness about this deadly infection. However, the lack in local antifungal surveillance data is jeopardizing the treatment of patients. In ICUs, any delay can leave only the option of intravenous amphotericin B which may have side–effects on the ICU patients due to kidney related problems or otherwise and cannot be administered or echinocandins which are only known to inhibit the growth. However, lacunae in date and surveillance render the extent of threat not yet completely known. When admitted the physiological scores can guide the intensivist for further action but when a fatal infection such as IA is suspected it is better to follow the local GM cut-offs for mortality, and initiate or change the therapy for the better outcome of patients. And also it would be in interests of the patients to have an active multi-azole susceptibility testing of *Aspergillus fumigatus* to monitor the extent of the problem and develop a local susceptibility data base which might aid in future treatments to limit the emergence of resistance.

## Figures and Tables

**Table 1:** Socio-demographic and underlying conditions of the patients enrolled in the study.

Variables	Total	Mortality in Invasive Aspergillosis (IA) CI: 0.23–0.35		*p*-Value	Unadjusted OR (95% CI)
		Survivor (*n* = 165; 70.2%)	Non-Survivors (*n* = 70; 29.7%)		
Age (years)		50.6 ± 15.8 (48.2–53.1)	50.7 ± 16.9 (46.7–54.8)	0.97	
0–12	6	2 (33.3)	4 (66.6)	0.23	
12–18	16	12 (75)	4 (25)		0.16 (0.02–1.29)
19–60	161	113 (70.1)	48 (29.8)		0.21 (0.03–1.19)
>60	52	38 (73)	14 (26.9)		0.18 (0.03–1.11)
Male	149	103 (69.1)	46 (30.8)	0.63	1.15 (0.64–2.07)
Seasonal variation					
Summer	66	39 (59)	27 (40.9)	0.07	2.15 (1.03–4.49)
Autumn	36	24 (66.6)	12 (33.3)		1.55 (0.64–3.76)
Hematological malignancy	27	12 (44.4)	15 (55.5)	0.002	3.47 (1.53–7.88)
CGD/TB	216	151 (69.9)	65 (30)	0.73	1.2 (0.41–3.48)
COPD	41	13 (31.7)	28 (68.2)	0.00	7.79 (3.71–16.35)
ARF	15	9 (60)	6 (40)	0.37	1.62 (0.55–4.75)
Metastatic cancer	3	2 (66.6)	1 (33.3)	0.89	1.18 (0.1–13.24)
Diabetes	14	6 (42.8)	8 (57.1)	0.02	3.41 (1.13–10.25)
Corticosteroid administration < 7 days	47	25 (53.1)	22 (46.8)	0.004	2.56 (1.32–4.96)
Corticosteroid administration ≥ 21 days	16	6 (37.5)	10 (62.5)	0.003	4.41 (1.53–12.68)

(OR, odds ratio; CI, confidence interval; CGD, chronic granulomatous disease; TB, tuberculosis; COPD, chronic obstructive pulmonary disease; ARF, acute renal failure; HIV/AIDS, human immunodeficiency virus infection and acquired immune deficiency syndrome).

**Table 2:** Clinical characteristics during ICU admission and stay (*n* = 235).

Variables	Total (*n* = 235)	Mortality in Invasive Aspergillosis (IA) CI: 0.23–0.35		*p*-Value	Unadjusted Odds Ratio (95% CI)
		Survivor (*n* = 165; 70.2%)	Non-Survivors (*n* = 70; 29.7%)		
Mechanical ventilation	95	63 (66.3)	32 (33.6)	0.28	1.36 (0.77–2.4)
Thrombocytopenia	85	58 (68.2)	27 (31.7)	0.61	1.15 (0.65–2.06)
Vasopressors	18	12 (66.6)	6 (33.3)	0.73	1.19 (0.42–3.32)
Pneumonia	80	55 (68.7)	25 (31.25)	0.58	1.11 (0.61–1.99)
Sepsis	19	12 (63.1)	7 (36.8)	0.48	1.41 (0.53–3.76)
Temperature				0.26	
38–39.3 °C	130	86 (66.1)	44 (33.8)		1.65 (0.9–3.04)
≥39.4 °C	16	11 (68.7)	5 (31.2)		1.47 (0.45–4.71
Prolonged treatments				0.003 F	
Steroids	26	10 (38.4)	16 (61.5)		3.76 (1.55–9.13)
HAART	3	2 (66.6)	1 (33.3)		1.17 (0.01–13.41)
Duration in ICU				0.88	
>4–7 days	69	46 (66.6)	23 (33.3)		1.31 (0.5–3.41)
>7–14 days	104	75 (72.1)	29 (27.8)		1.01 (0.4–2.54)
>14 days	33	23 (69.7)	10 (30.3)		1.14 (0.37–3.43)
Neutropenia	35	22 (62.8)	13 (37.1)	0.302	1.48 (0.69–3.14)
Malnourishment	10	4 (40)	6 (60)	0.03	3.77 (1.03–13.81)
Mechanical ventilation	192	61 (66.3)	31 (33.7)	0.29	1.35 (0.76–2.39)
Dialysis	13	6 (46.1)	7 (53.8)	0.05	2.94 (0.95–9.1)
Antibiotics administered					
Cefoperazone-sulbactam	113	80 (70.8)	33 (29.2)	0.85	0.94 (0.54–1.65)
Amikacin	153	114 (74.5)	39 (25.4)	0.04	0.56 (0.31–1)
Piperacillin-tazobactam	74	42 (56.7)	32 (43.2)	0.002	2.46 (1.37–4.43)
Vancomycin	90	66 (73.3)	24 (26.6)	0.41	0.78 (0.43–1.4)
Linezolid	71	49 (69)	22 (30.9)	0.79	1.08 (0.59–1.98)
Metrogyl	62	52 (83.8)	10 (16.1)	0.00	0.36 (0.17–0.76)
Meropenam	107	75 (70)	32 (29.9)	0.97	1.01 (0.57–1.77)
Levofloxacin	91	65 (71.4)	26 (28.5)	0.74	0.9 (0.51–1.61)
Other β lactam drugs	26	21 (80.7)	5 (19.21)	0.21	0.52 (0.19–1.46)
Radiological findings				0.86	
Pleural effusion	10	6 (60)	4 (40)		1.47 (0.39–5.16)
Consolidation	10	6 (60)	4 (40)		1.47 (0.39–5.6)
*Aspergillus* culture positivity	34	7 (20.59)	27 (79.41)	0.00	14.17 (5.77–34.75)
Galactomannan cut-off for mortality (≥1.04)	64	12 (18.75)	52 (81.25)	0.00	36.83 (16.62–81.59)

(ANC, absolute neutrophil count; HAART, highly active antiretroviral therapy; GGO, ground glass opacity).

**Table 3:** Galactomannan values using AspICU diagnostic criteria and the outcomes.

Survival within 30 Days	Total (*n* = 235)	AspICU Criteria for Invasive Aspergillosis (IA)			*p*-Value	OR (95% CI)	
		Proven + Putative (*n* = 22; 9.36%) (mean ± SD: 2.46 ± 1.75; median: 1.64)	No IA (*n* = 201; 85.5%) (mean ± SD: 0.83 ± 0.5; median: 0.7)	Colonization (*n* = 12; 5.1%) (mean ± SD: 4.83 ± 1.65; median: 5.79)		Unadjusted	Adjusted
Survivor *n* (%); Median	165	6 (3.6); 1.47	158 (95.7); 0.57	1 (0.6); 1.09	0.00		
Non-survivor *n* (%); Median	70	16 (22.8); 2.09	43 (61.4); 1.48	11 (15.7); 6		14.17 (6.7–34.75)	1.34 × 10^17^

(F, Fisher’s exact test; OR, odds ratio; CI, confidence interval; SD, standard deviation).

**Table 4:** Univariate and multivariate analysis of the severity scores obtained by three ICU scoring systems and galactomannan antigen values.

Variable	Total (*n* = 235)	Mortality in Invasive Aspergillosis (IA) CI: 0.23–0.35		*p*-Value	OR (95% CI)	
		Survivor *n* = 165	Non-Survivors *n* = 70		Unadjusted	Adjusted
APACHE II score Mean ± SD; median (95% CI)	235 15.81 ± 4.25; 16 (15.23–16.36)	165 15.35 ± 4.23; 15 (14.7–16)	70 16.88 ± 4.14; 17 (15.89–17.87)	0.011	1.08 (1.01–1.16)	1.13 (0.99–1.29)
SAPS II score Mean ± SD; median (95% CI)	216 35.82 ± 7.68; 35 (34.79–36.58)	153 35 ± 7.6; 35 (33.8–36.2)	63 37.6 ± 7.5 (35.7–39.5)	0.022	1.04 (1–1.08)	0.98 (0.90–1.06)
SOFA score Mean ± SD; median (95% CI)	235 6.71 ± 1.49; 7 (6.51–6.9)	165 6.52 ± 1.47; 7 (6.3–6.75)	70 7.14 ± 1.45; 7 (6.76–7.49)	0.003	1.32 (1.09–1.06)	0.91 (0.90–1.06)
GM values Mean ± SD; median (95% CI)	235 1.19 ± 1.25; 0.79 (1.03–1.35)	165 0.68 ± 0.34; 0.606 (0.63–0.73)	70 2.37 ± 1.73; 1.64 (1.96–2.79)	0.000	44.61 (15.71–126.69)	56.57 (17.25–185.44)

(SD, standard deviation; OR, odds ratio; CI, confidence interval; APACHE II, Acute Physiology and Chronic Health Evaluation II; SOFA, Sequential Organ Failure Assessment; SAPS II, Simplified Acute Physiology Score II; GM, galactomannan antigen).

**Table 5:** Area under the receiver operating characteristic (ROC) curve, Hosmer–Lemeshow goodness-of-fit statistics, and predicted and standardized mortality ratios.

ICU Mortality Scores	ROC Curve		Hosmer–Lemeshow Goodness-of-Fit		Predicted Mortality, %	Standardized Mortality Ratio (95% CI)
	AUC, (SE)	95% CI	χ2	*p*		
APACHE II	0.595 (0.03)	(0.518–0.672)	11.04	0.92	24.63	1.20 (0.94–1.51)
SAPS II	0.602 (0.04)	0.518–0.686)	38.69	0.13	20.07	1.46 (1.13–1.86)
SOFA	0.610 (0.03)	(0.534–0.685)	9.49	0.14	14.89	2.00 (1.57–2.51)
GM values	0.924 (0.01)	(0.888–0.960)	154.21	0.52	29.78	1 (0.78–1.51)

(ROC, receiver operating curve; AUC, area under the curve; SE, standard error; CI, confidence interval; APACHE II, Acute Physiology and Chronic Health Evaluation II; SOFA, Sequential Organ Failure Assessment; SAPS II, Simplified Acute Physiology Score II; GM, galactomannan antigen).

**Table 6:** Concordance and intraclass coefficients (ICC) between CLSI M38-A2 and EUCAST guidelines.

Antifungal	CLSI (%) (*n*, 34)		EUCAST (%) (*n*, 34)		Concordance	ICC * (95% CI)
	Susceptible	Non-Susceptible	Wild-Type	Non Wild-Type		
Itraconazole	25 (73.52%)	9 (26.47%)	24 (70.58%)	10 (29.41%)	99	0.97 (0.93–0.99)
Voriconazole	33 (97.05%)	1 (2.94%)	33 (97.05%)	1 (2.94%)	94	0.96 (0.91–0.98)
Posaconazole	33 (97.05%)	1 (2.94%)	31 (91.17%)	3 (8.82%)	91	0.91 (0.69–0.97)
Amphotericin B	29 (95.29%)	5 (14.70%)	29 (95.29%)	5 (14.70%)	98	0.97 (0.76–0.99)
Caspofungin	34 (100%)	0	33 (97.05%)	1 (2.94%)	90	0.94 (0.88–0.97)
Micafungin	34 (100%)	0	34 (100%)	0	100	1

(* ICC, intraclass coefficient).

**Table 7:** Characteristics associated with mutations detected in azole-resistant *Aspergillus fumigatus*.

GenBank Accession Number	Sex, Age (Years)	Underlying Disease	Clinical Specimen	AspICU Criterion	Delta (∆) GM (∆GM = GM1_day 0_-GM2 _day 7_)	Type of Mutation	Itr MIC (µg/mL)	Vor MIC (µg/mL)	Pos MIC (µg/mL)	Resistant Antifungal	Antifungal Treatment	30-Day Outcome
MF148156	M, 45	COPD, TB	Sputum	Putative	−0.46	G54R (hotspot)	8	4	0.03	ITR, VOR	AMB	Died
MF148157	M, 49	COPD, TB	Sputum	Putative	0.29	G54R (hotspot)	4	0.06	0.03	ITR	AMB	Died
MF148158	M, 48	TB	BAL, pleural fluid	Putative	−0.35	G54R (hotspot)	8	0.06	0.03	ITR	AMB	Died
MF148159	M, 67	COPD, TB	ETA	Putative	−0.965	P216L (defined)	16	0.06	1	ITR, POS	AMB+ VOR	Died
MF148160	F, 59	COPD, TB	BAL	Putative	0.829	P216L (defined)	8	0.03	0.03	ITR	VOR	Survived
MF148161	M, 45	TB	Sputum	Colonizer	−2.08	G54R (hotspot)	16	0.06	0.03	ITR	AMB	Died

(M, male; F, female; COPD, chronic obstructive pulmonary disease; TB, tuberculosis; BAL, bronchoalveolar lavage; ETA, endotracheal aspirate; P, proline; L, leucine; G, glycine; R, arginine; MIC, minimum inhibitory concentration; ITR, itraconazole; VOR, voriconazole; POS, posaconazole; AMB, amphotericin B).

## Supplementary Materials

The following are available online at https://www.mdpi.com/article/10.3390/jof7110991/s1, Table S1: Sample and culture distribution, Table S2: Summary of few studies depicting hot-spot and other mutations in cyp51A gene of *A. fumigatus* imparting resistance to azole drugs.
