# Supplementary material for: Prognostic Scores and Azole-Resistant Aspergillus fumigatus in Invasive Aspergillosis from an Indian Respiratory Medicine ICU (ICU Patients with IA Suspicion)
Source: J Fungi (Basel). 2021 Nov 20;7(11):991. doi: 10.3390/jof7110991 (PMC8625311; doi:10.3390/jof7110991)
Supplement: Supplementary file 1 [file jof-07-00991-s001.zip › jof-1437679-supplementary.pdf]

Table S1. Sample and culture distribution.

Sample	Total patients/ samples	Direct microscopy positive	Culture positive	Repeat isolation
Blood	235/ 449	Not applicable	*1 (A.nidulans)*	*1 (A.nidulans)*
Sputum	188/ 197	21	*20 (12 A. flavus, 8 A.fumigatus)*	*6 (4 A.flavus, 2 A.fumigatus)*
BAL	24/ 41	7	*7 (1 A.flavus, 4 A.fumigatus, 2 A.terreus)*	*6 (1 A.flavus, 4 A.fumigatus, 1 A.terreus)*
ETA	23/ 39	5	*5 (2 A.flavus, 2 A.fumigatus, 1 A.niger)*	*5 (2 A.flavus, 2 A.fumigatus, 1 A.niger)*
Pleural effusion	5/ 5	1	*1 (1 A.fumigatus) (similar isolation from patient's sputum sample)*	
Total	235/ 731	33	*34 (9 A.flavus, 10 A.fumigatus, 2 A.terreus, 1 A.niger, 1 A.nidulans) (1 patient had same isolate with two samples)*	*18*

[Note: BAL: bronchoalveolar lavage; ETA: endotracheal aspirate]

Table S2. Summary of few studies depicting hot-spot and other mutations in cyp51A gene of *A. fumigatus* imparting resistance to azole drugs.

Total no. of *A. fumigatus*, source and drug resistant	Mutations in cyp51A	No of isolates showing mutations	Breakpoints followed	Methodology and Endpoint	Country/ Region	Reference
12(clinical): ITRA	Codon 54: G54E, G54V, G54R; Codon 236: M236T, M236V, M236K	6	≤0.5µg/ml susceptible and >8 resistant	CLSI NCCLS Endpoint: Complete inhibition of growth	Madrid, Spain/ Europe	Diaz-Guerra Tm et al., 2003

130(clinical): ITRA	TR34, L98H;<br>S297T, F495I;<br><br>G54E/W, G138C, M220R/I/V/K	105;<br>12;<br><br>8	itraconazole and voriconazole: <2 mg/l (susceptible), 2mg/l (intermediate), >2 mg/l (resistant); posaconazole <0.25, 0.5 and >0.5 mg/l	Agar screening: 4/8 mg/l itraconazole in SDA<br><br>CLSI M38-A	Nijmegen, Netherlands/ Europe	Snelders E et al., 2010
121 (clinical) ITRA, VOR, and POS	TR34/L98H	5	resistant: >2mg/l, itraconazole: and voriconazole and >0.25mg/l, posaconazole	EUCAST	Poland/ Europe	Nawrot U et al., 2017
3(environmental): Itraconazole<br>1: VOR<br>1: POS	TR34, L98H	4	susceptible for itraconazole and voriconazole≤1mg/l;<br>posaconazole≤0.25 mg/l	Agar screening: 4 mg/l itraconazole in SDA<br>EUCAST<br>Endpoint: No visible growth	Copenhagen, Denmark/ Europe	Mortensen KL et al., 2010
1(clinical):Pan azole	G432S	1	resistance cut-offs for itraconazole and voriconazole: >2mg/l; posaconazole >0.25mg/l	ETest	Paris, France/ Europe	Alanio A et al., 2011
14(clinical): ITRA	TR34, L98H	14	Not applicable	CLSI M38-A	Nijmegen, Netherlands/ Europe	Mellado E et al., 2007
17(clinical): ITRA	TR34/L98H<br>M220I<br>G54W<br>F219C	6<br>1<br>1<br>1	resistance cut-offs for itraconazole and voriconazole: >2mg/l; posaconazole >0.25mg/l	EUCAST<br>Endpoint: No visible growth	Germany/ Europe	Bader O et al., 2013
1(clinical): ITRA and VOR	TR46/Y121F/<br>T289A	1	Not applicable	EUCAST	France/ Europe	Lavergne R-A et al., 2015
1(clinical): ITRA and VOR	TR46/Y121F/<br>T289A	1	Not applicable	Sensititre YeastOne YO10; CLSI M38-A2	Belgium/ Europe	Montesinos I et al., 2014
4(clinical): Pan azole	TR46/Y121F/T298A ; M172I, G448S<br>M220R<br>TR34/L98H	1<br><br>1<br>1	resistance cut-offs for itraconazole and voriconazole: >2mg/l; posaconazole >0.25mg/l	EUCAST	Nijmegen, Netherlands/ Europe	van Ingen J et al., 2015

21(clinical): ITRA and VOR	TR46/Y121F/ T289A<br>TR34/L98H<br>M172I/G448S<br>S297T/F495I	3<br><br>15<br>1<br>2	voriconazole: resistant: >2mg/l, susceptible: ≤1 mg/l.<br>itraconazole: resistant: >2mg/l, susceptible: ≤1 mg/l.<br>posaconazole: resistant: >0.25mg/l, susceptible: ≤0.12 mg/l.	EUCAST	Nijmegen, Netherlands/ Europe	Fuhren J et al., 2015
0 (clinical/hospital environment)	N479D;F165L;N248K;D262Y;M172V;F46Y;E427K;D255E; N248T; Other silent mutations	9	resistant: ≥4 mg/l susceptible:<1 mg/l	CLSI M38-A2	Madrid, Spain/ Europe	Escribano P et al., 2011
220 (clinical) ITRA, VOR, and POS	M220V,TR46/Y121F/T289A, G54R, G138S, TR34/L98H, G448S, F219S, M220I, G54E, G138C	26	resistant: voriconazole and itraconazole ≥4 mg/l; posaconazole ≥0.5 mg/l	CLSI M38-A2	USA/ North America	Wiederhold NP et al., 2016
0	N248K;<br>I242V;<br>A9T;<br>A9T	5	resistance cut-offs for itraconazole and voriconazole: >2mg/l	Sensititre YeastOne YO10; CLSI M38-A2<br>Endpoint: Complete inhibition of growth	Toronto, Canada/ North America	Shalhoub S et al., 2015
38 (environmental)	TR46/Y121F/T289A, TR53, TR34/L98H	17	Not applicable	CLSI M38-A2	Bogotá, Colombia/ Latin America	Le Pape P et al., 2016
1 (clinical) ITRA	G54E	1 (fungal keratitis case)	Not applicable	CLSI M38-A2	Argentina/ South America	Leonardelli F et al., 2017
4(clinical): ITRA	M220I;<br>G54R	1;<br>3	Not applicable	E-Test<br>CLSI M38-A<br>Endpoint: No visible growth	Beijing, China/ Asia	Chen J et al., 2005
38 (clinical)<br>ITRA, VOR, and POS	TR34/L98H	3	resistant: >1, >1 or >0.5 mg/l to itraconazole, voriconazole and posaconazole, respectively	Sensititre YeastOne, CLSI M38-A2	Taiwan/ Asia	Wu CJ et al., 2015

50 (environmental), 16 (clinical)<br>ITRA, VOR, and POS	TR34/L98H	1 (environmental), 2 (clinical)	resistant: ≥2, ≥0.5 and ≥2 mg/l to itraconazole, posaconazole and voriconazole, respectively	E-test, CLSI M38-A2	Kuwait/ Middle East	Ahmad S et al., 2015
746 (clinical)<br>ITRA, VOR, and POS	TR34/L98H	66 mutations detected; 10 undetected	Not applicable	CLSI M38-A	Turkey/ Middle East	Özmerdiven GE et al., 2015
418 (clinical)<br>ITRA, VOR, and POS	TR34/L98H, G54R, F46Y, Y431S and G448S	6 mutations detected; 3 undetected	resistant: >2mg/l, itraconazole: and voriconazole and >0.25mg/l, posaconazole	CLSI M38-A2	Australia/ Oceania	Kidd SE et al., 2015
685 (clinical)<br>ITRA, VOR, ISA and POS	(10) TR34/L98H, (1) G54E and one isolate with non-synonymous mutation	12	itraconazole, 1 µg/ml; voriconazole, 1 µg/ml; posaconazole, 0.5 µg/ml; isavuconazole, 1 µg/ml; amphotericin B, 4 µg/ml and caspofungin, 0.25 µg/ml	CLSI M38-A2	Delhi, India/ Asia	Chowdhary A et al., 2015

[NOTE: ITR: itraconazole; VOR: voriconazole; POS; posaconazole; ISA: isavuconazole; CLSI: Clinical and Laboratory Standards Institute; EUCAST: European Committee on Antimicrobial Susceptibility Testing]
